# *Toxoplasma gondii* as a drug for anti-tumor immunotherapy: mechanisms, challenges, and perspectives

**DOI:** 10.1051/parasite/2026006

**Published:** 2026-02-05

**Authors:** Jing Li, Eman E. El Shanawany, Soad E. Hassan, Peng-Yao Li, Jia-Hui Sun, Hong-Mei Li, Shao-Hong Lu, Xiao-Nong Zhou, Bin Zheng

**Affiliations:** 1 School of Basic Medicine and Forensic Medicine, Zhejiang Key Laboratory of Biosafety and Biomedical Translation, Zhejiang Industrial Technology Engineering Center for Novel Vaccines, Hangzhou Medical College Xihu District Hangzhou 310013 PR China; 2 Parasitology and Animal Diseases Department, Veterinary Research Institute, National Research Centre Dokki Giza 126 Egypt; 3 College of Medical laboratory Techniques, Al-Farahidi University Baghdad 1001 Iraq; 4 National Key Laboratory of Intelligent Tracking and Forecasting for Infectious Diseases, National Institute of Parasitic Diseases, Chinese Center for Disease Control and Prevention (Chinese Center for Tropical Diseases Research), NHC Key Laboratory for Parasitology and Vector Biology, WHO Collaborating Centre for Tropical Diseases, National Centre for International Research on Tropical Diseases 207 Ruijin 2nd Road Huangpu District Shanghai 200025 PR China; 5 Hainan Tropical Diseases Research Center (Hainan Sub-Center, Chinese Center for Tropical Diseases Research) Longhua District Haikou 570203 PR China

**Keywords:** *Toxoplasma gondii*, Anti-tumor, Immunity

## Abstract

*Toxoplasma gondii* is an intracellular protozoan parasite known to infect a wide range of hosts, including humans, and is a significant cause of health issues, particularly in pregnant women and immunocompromised individuals. However, it has garnered attention for its potential in cancer treatment due to its diverse anti-cancer mechanisms. *Toxoplasma gondii* induces key cytokines such as IL-12 and IFN-γ, triggering robust Th1 immune responses that effectively target tumor cells. Furthermore, it modulates the immunosuppressive tumor microenvironment (TME), reduces inhibitory immune cells, promotes activated immune cells, induces apoptosis in tumor cells, inhibits proliferation, and disrupts tumor angiogenesis through regulatory signaling pathways. Despite these promising antitumor attributes, significant limitations hinder its translation into clinical practice. These include strain-dependent differences in virulence and therapeutic efficacy, ethical and biosafety concerns associated with wild-type strains, limited applicability of animal data to human therapy, and the possibility that the parasite may promote tumorigenesis under certain conditions. Innovative approaches such as engineered strains for precise tumor targeting, exploitation of its bioactive agents, use as a drug carrier for brain tumors, and combination therapies with other anti-cancer modalities show promise. These advances, coupled with comprehensive cost-effectiveness assessments, present new opportunities and hope for integrating *T. gondii* into cancer therapy.

## Abstract (fr)

*Toxoplasma gondii* est un protozoaire parasite intracellulaire connu pour infecter un large éventail d’hôtes, y compris l’homme, et constitue une cause importante de problèmes de santé, notamment chez les femmes enceintes et les personnes immunodéprimées. Cependant, son potentiel dans le traitement du cancer, grâce à ses divers mécanismes d’action anticancéreux, suscite un intérêt croissant. *Toxoplasma gondii* induit la production de cytokines clés telles que l’IL-12 et l’IFN-γ, déclenchant de fortes réponses immunitaires Th1 qui ciblent efficacement les cellules tumorales. De plus, il module le microenvironnement tumoral immunosuppresseur (TME), réduit le nombre de cellules immunitaires inhibitrices, favorise l’activation des cellules immunitaires, induit l’apoptose des cellules tumorales, inhibe la prolifération et perturbe l’angiogenèse tumorale par le biais de voies de signalisation régulatrices. Malgré ces propriétés antitumorales prometteuses, d’importantes limitations entravent son application clinique. Ces difficultés incluent des différences de virulence et d’efficacité thérapeutique selon les souches, des préoccupations éthiques et de biosécurité liées aux souches sauvages, une applicabilité limitée des données animales à la thérapie humaine et la possibilité que le parasite favorise la tumorigénèse dans certaines conditions. Des approches innovantes, telles que des souches modifiées pour un ciblage tumoral précis, l’exploitation de ses agents bioactifs, son utilisation comme vecteur de médicaments pour les tumeurs cérébrales et les thérapies combinées avec d’autres modalités anticancéreuses, sont prometteuses. Ces avancées, associées à des évaluations complètes du rapport coût-efficacité, offrent de nouvelles perspectives et un espoir pour l’intégration de *T. gondii* dans le traitement du cancer.

## Introduction

Cancer is a complex group of diseases characterized by uncontrolled cell growth and metastasis, and it ranks as the second leading cause of death, following cardiovascular diseases, in developing countries [96]. While the immune system can prevent metastasis, in some cases, it fails to effectively eliminate tumors [25]. Traditional treatments like surgery and chemotherapy have significant drawbacks [64]. Immunotherapy, which stimulates the immune system to target and destroy cancer cells, offers new hope [20, 72]. It is more specific and less toxic than traditional methods, enhancing the immune system’s natural ability to fight cancer [34, 47, 60]. However, its effectiveness varies among patients, and research is ongoing to improve outcomes [58].

Microorganism-based immunotherapy is an innovative cancer treatment that leverages the unique properties of bacteria, viruses, and parasites to modulate the immune system and target tumors. Various pathogens, including bacteria [21, 67, 113], viruses [39, 40], and parasites like *Trypanosoma cruzi* [81], *Echinococcus granulosus* [80], and *Trichinella spiralis* [82], have demonstrated the ability to stimulate immune responses that can eradicate tumor cells.

One promising parasite is *Toxoplasma gondii*, which has garnered attention for its potential in cancer immunotherapy [51]. Unlike bacteria and viruses, protozoa, as eukaryotes, can precisely express human proteins and tumor antigens, thereby stimulating targeted anti-tumor immunity within the host and enhancing the efficacy of tumor-specific treatment, which is a key requirement for effective anti-tumor therapy [112].

*Toxoplasma gondii*, an obligate intracellular protozoan parasite, releases a variety of effector proteins after invading host cells. These effectors modulate host cell signaling pathways, manipulate apoptotic processes, and hijack host-derived nutrients to support parasite survival, thereby facilitating its replication, dissemination, and persistence within the host. Recent studies have demonstrated that specific antigens and effector proteins of *T. gondii* can suppress the proliferation, invasion, and metastasis of multiple cancer types [84]. Importantly, *T. gondii* infection simultaneously activates both innate and adaptive immune responses, significantly influencing anti-tumor immunity [18, 86]. It may also limit tumor spread through mechanisms like hypoxia, avascular necrosis, anti-angiogenesis, and Th1 immune activation [15, 44]. Despite ongoing challenges related to virulence and ethical concerns in tumor therapy, substantial progress has been achieved in recent years in developing engineered *T. gondii* strains and identifying its intrinsic anti-tumor factors. These advances have further revealed the great potential of *T. gondii* in treating tumors [22].

This review summarizes recent advances in understanding the anti-tumor properties of *T. gondii*, including the mechanisms underlying its anti-cancer effects, the parasite’s effector molecules that activate host anti-tumor immunity, and its potential as a vector for brain tumor therapeutics and drug delivery. It also addresses the obstacles encountered in *T. gondii* anti-tumor research and future research directions.

## Mechanisms underlying the anti-tumor effects of *T. gondii*

**Figure 1:**
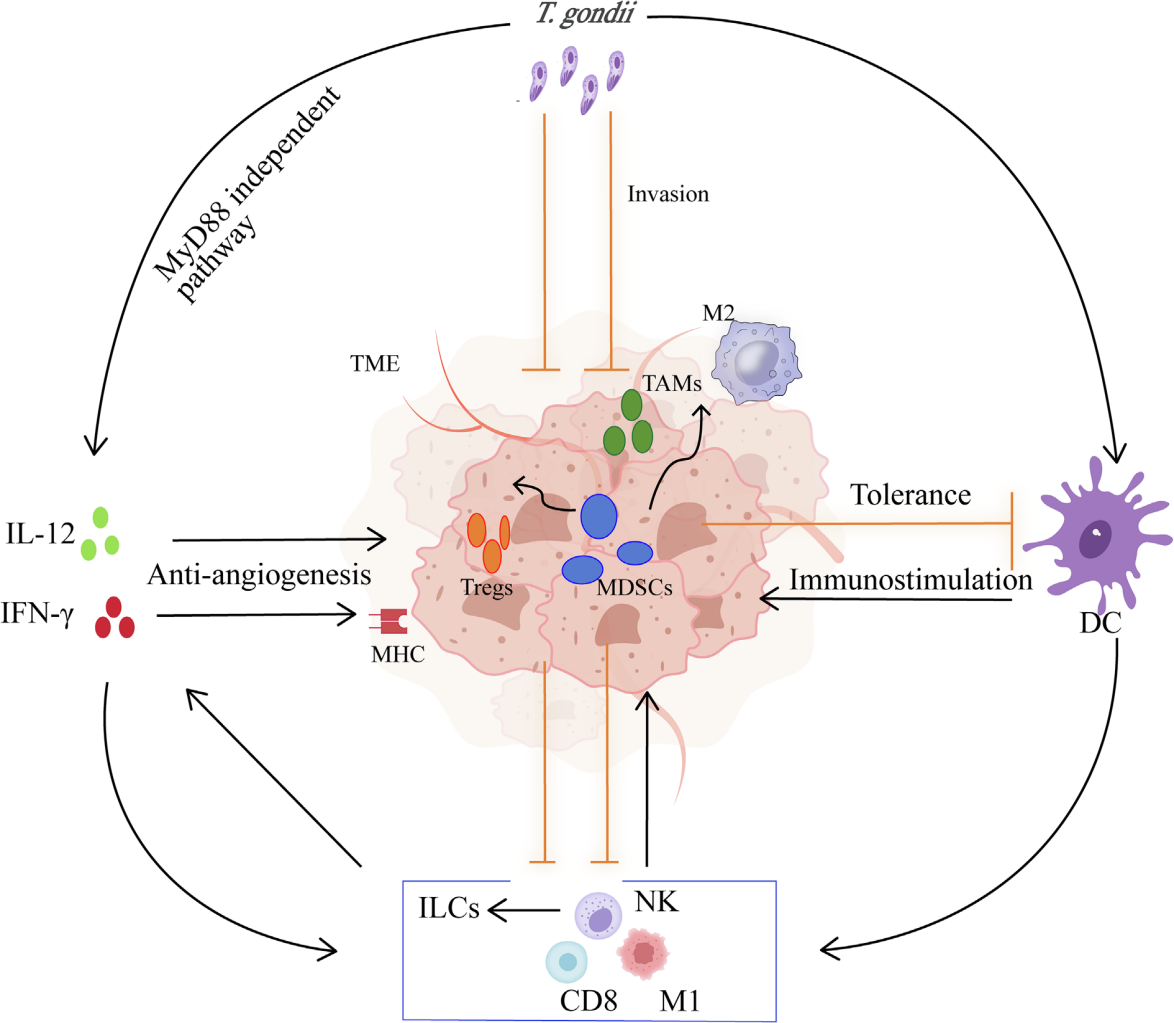
Mechanisms underlying the anti-tumor effects of *T. gondii.*

*Toxoplasma gondii* has demonstrated potential as a novel immune-modulating agent for cancer therapy. By leveraging its unique ability to interact with the immune system and the tumor microenvironment (TME) (Fig. 1), *T. gondii* can i) induce immune activation, ii) modulate the immunosuppressive nature of tumors, and iii) directly affect tumor cells.

*Toxoplasma gondii* triggers a Th1-type immune response *via* the IL-12/IFN-γ axis through MyD88-dependent and MyD88-independent pathways. It activates DCs and MDSCs, promotes the secretion of IFN-γ by T cells, NK cells, and ILCs, induces the polarization of M2 macrophages to M1 macrophages, enhances cytotoxicity, inhibits angiogenesis, induces apoptosis of malignant cells, and reverses the tumor microenvironment. CD8, cytotoxic T lymphocyte; DC, dendritic cell; IFN-γ, interferon-gamma; IL-12, interleukin-12; ILCs, innate lymphoid cells; M1/M2, classically or alternatively activated macrophages; MDSCs, myeloid-derived suppressor cells; MHC, major histocompatibility complex; NK, natural killer cell; TAMs, tumor-associated macrophages; TME, tumor microenvironment; Tregs, regulatory T cells.

### Activation of innate and adaptive immune responses by *T. gondii*

The parasite primarily targets myeloid cells (*e.g.*, dendritic cells and macrophages), promoting their maturation and enhancing antigen presentation, thereby activating T cells [68]. Innate immunity mainly drives CD11c^+^ dendritic cells and macrophages to increase the expression of various inflammatory factors, such as IL-12 through the TLR/MyD88 pathway, and induces a Th1-type immune response *via* the IL-12/IFN-γ axis [12, 36]. This in turn stimulates CD4^+^ and CD8^+^ T cells, NK cells, and ILCs to secrete IFN-γ, polarizes macrophages toward the M1 phenotype, initiates adaptive immunity [32], and recruits neutrophils as well as inflammatory monocytes to the infection site. In addition, in *T. gondii*-infected MyD88^−/−^ mice or cells, it can still induce the expression of partial IL-12p40 and chemokines (CCL17/22), suggesting the existence of an MyD88-independent pathway [92]. The *T. gondii* dense granule protein GRA24 phosphorylates p38 MAPK and activates the production of IL-12. Furthermore, the *T. gondii*-secreted protein Cyclophilin-18 (TgCyp18) couples with CCR5-GiPCR and induces IL-12p40 through the Gαi-PI3K-ERK pathway [46, 61]. This dual activation mechanism of innate immunity and adaptive immunity lays the foundation for the development of anti-tumor immunotherapy strategies.

### *Toxoplasma gondii* modulation of the TME, induction of tumor cell apoptosis, and inhibition of angiogenesis

The TME is typically characterized by immunosuppression, primarily driven by MDSCs, Tregs, and TAMs, which collectively fuel tumor growth and metastasis. As tumors progress, MDSCs expand massively and play a pivotal role in tumor development, metastasis, and therapeutic resistance, making them a promising therapeutic target in cancer treatment [26, 56]. *Toxoplasma gondii* possesses the characteristic of preferentially infecting myeloid cells and achieving immune evasion *via* the “Trojan horse” mechanism. Specifically, *T. gondii* preferentially infects MDSCs, prompting these immunosuppressive cells to transform into an immunostimulatory phenotype and thereby enhancing antigen presentation efficiency [18]. During this process, *T. gondii* can stimulate robust Th1 immune responses and CD8^+^ T cell responses *via* TLR/MyD88, Gαi-PI3K-ERK, and GRA24-p38 MAPK signaling pathways, which in turn strengthens the tumor-killing capacity of NK cells and CD8^+^ T cells. Simultaneously, it upregulates the expression of iNOS/ROS, promoting the polarization of TAMs/MDSCs from the M2 type to the M1 type. Additionally, it reduces the population of Foxp3^+^ regulatory T cells and suppresses VEGF secretion, thereby converting immunologically “cold” tumors into highly active “hot” TMEs [10, 38, 57, 88]. The molecular signaling pathways modulated by *T. gondii* largely overlap with those disrupted during tumorigenesis. On the one hand, viable and invasive *T. gondii* is essential for activating the IL-12/IFN-γ axis and driving robust CD4^+^ and CD8^+^ T cell responses [36]. On the other, both before and after *T. gondii* invades host cells, it secretes rhoptry proteins (ROPs), microneme proteins (MICs), and dense granule proteins (GRAs); some of these have been proven to possess anti-tumor effects by reversing immune suppression [36].

*Toxoplasma gondii* directly assaults tumor cells by inducing apoptosis, blocking proliferation, and curbing migration. Its cytokine network, characterized by TNF-α and IFN-γ, synergistically enhances the expression of pro-apoptotic molecules such as Bax and caspases, triggering mitochondria-dependent apoptosis, while upregulating p21 and p27 to halt the cell cycle at the G1 phase [85]. The parasite also forces cancer cells into G2/M stalling *via* cyclin B1, and further amplifies apoptosis *via* the p53-Bax-caspase-3/9 cascade, accompanied by Ki-67 down-regulation and classic apoptotic body formation. Additionally, *T. gondii* increases E-cadherin expression in breast cancer MDA-MB-231 cells, markedly reducing cell motility and thus blocking tumor progression on multiple fronts [1, 71, 105]. GRA16 can inhibit NF-κB activation by regulating the PP2A-B55/AKT/NF-κB signaling pathway, making tumor cells sensitive to apoptosis induced by chemotherapeutic drugs [87]. To achieve immune escape characteristics, *T. gondii* also inhibits apoptosis in myeloid cells [54]. This dual role of promoting apoptosis in tumor cells, while inhibiting it in immune cells, may enhance the potential of *T. gondii* in tumor treatment by activating the immune response against tumors.

Additionally, *T. gondii* has the ability to inhibit tumor angiogenesis. In the B16F10 melanoma model, *T. gondii* infection severely restricts angiogenesis by inducing hypoxia and avascular necrosis, effectively inhibiting tumor growth [44]. Exosomes derived from *T. gondii*-infected dendritic cells further inhibit angiogenesis in colorectal cancer models by altering the TME, thereby limiting tumor progression [114]. During the activation of the IL-12/IFN-γ pathway by *T. gondii* infection, IL-12 shows anti-angiogenic effects by suppressing the mRNA expression of VEGF, bFGF, and MMP-9, while IFN-γ promotes apoptosis and inhibits the proliferation of human tumors by secreting the anti-angiogenic chemokine IP-10 [50]. IFN-γ directly suppresses the transcriptional activity of HIF-1α *via* the JAK-STAT1 axis, and overexpression of STAT1 blocks hypoxia-induced up-regulation of vascular endothelial growth factor VEGF-A [106, 108].

## Limitations of the anti-tumor effect of *T. gondii*

### Diversity of *T. gondii* strain and variations in infection patterns

The protozoan has been shown to inhibit melanoma, ovarian cancer, lung cancer, and other types of cancer. Different strains of *T. gondii* tend to show differential anti-tumor effects. Although they have highly similar genetic backgrounds, classical typing of *T. gondii* strains divides them into three major lineages (I, II, and III), with virulence being the most pronounced difference: type I strains (*e.g.*, RH and GT1) are highly virulent, whereas type II and III strains (*e.g.*, Pru, ME49, CTG, and VEG) are of low virulence. With the advent of high-throughput genomic technologies, numerous recombinant or atypical genotypes have been identified. A clustering-based study integrated the marked genetic diversity of 138 unique genotypes into 15 haplotype groups, further summarized into six major lineages that systematically describe the evolutionary subtypes of *T. gondii* [83, 90]. Beyond virulence, different strains also vary in migratory capacity, persistence of infection, and anti-tumor efficacy, adding complexity to their use as live therapeutic platforms [31]. Both RH and ME49 strains can reduce the growth and migration of breast cancer cells by altering the transcriptional regulation of signaling pathways involving genes such as BRCA1, MYC, and IL-6. However, compared to ME49, infection with the RH strain in human breast cancer cells (MDA-MB-231) results in higher E-cadherin expression, which inhibits cancer cell migration [105]. Oral infection with the ME49 strain of *T. gondii* can induce Th1 immune responses and anti-angiogenic activity, inhibiting the growth of Lewis lung cancer. Following infection with the ME49 strain, there was a marked enhancement in the survival rate, the proportion of CD8^+^ T cells, the expression levels of IFN-γ mRNA, the titers of IgG2a in the serum, and CTL response in Lewis lung cancer model mice [52]. The Type II Pru strain has contrasting effects on the development of Lewis lung cancer. Acute infection with Pru restricts tumor growth, while chronic Pru infection increases the rate of tumor formation [89].

Selecting the most suitable strain is theoretically feasible, but it is restricted by multiple practical conditions. The expression levels of functional genes vary among different strains. There are differences in the intensity of strain-specific immune stimulation of the GRAs in *T. gondii*. The polymorphism of GRA15 in the three clonal linetions of *T. gondii* in North America enables the *T. gondii* type II strain to activate the NF-κB pathway much more effectively than the type I and type III strains. The different immune responses triggered lead to different tumor effects [62, 78]. In addition, the TME and immune capacity of different patients directly affect the activation effect of *T. gondii* on immune pathways, leading to differences in therapeutic efficacy of the same strain in different individuals [85].

### The risk of *T. gondii* virulence

Since *T*. *gondii* is a parasitic protozoan, its use raises ethical controversies when its wild-type strains are applied directly for anti-tumor treatment in the human body. In recent years, significant progress has been made in the research on attenuated strains of protozoa.

*Toxoplasma gondii* replication-deficient attenuated strains have shown potential in tumor therapy by activating immune responses, enhancing anti-tumor immunity, and synergizing with immune checkpoint inhibitors, providing new insights and strategies for cancer immunotherapy. The uracil auxotroph strain of *T. gondii* (RH-Δ*CPS*) can convert immunosuppressive ovarian cancer-associated CD45^+^CD11c^+^ cells into immunostimulatory cells, upregulate costimulatory molecules CD80 and CD86 to restore antigen-presenting capabilities, and activate CD8^+^ T cell responses [5]. In addition, it can activate systemic anti-tumor immune activity and memory, causing the regression of established B16F10 mouse melanoma [4]. *Toxoplasma gondii* ME49Δ*GRA5* upregulates Th1 cytokines and tumor-infiltrating T cell levels in the TME, reducing the growth of mouse breast cancer (4T1) and preventing lung metastasis [19]. The uracil auxotrophic *T. gondii* RH strain (RH-Δ*ompdc*) can also inhibit the growth of 4T1 breast cancer tumors and lung metastasis [102]. The ME49Δ*ompdc/gra4* strain of *T. gondii* is capable of eliciting an enhanced type I interferon (IFN-I) response and specifically enriching for a subset of dendritic cells characterized by the phenotype CD64^+^MAR-1^+^CD11b^+^, which in turn augments T cell-mediated anti-tumor immunity [43].

Although numerous studies have indicated that *T. gondii* knockout strains can stimulate the immune system and may play a role in future cancer treatments, most of these investigations are still in their early stages. There is a lack of clinical data assessing the safety of virulence in immunotherapeutic interventions, and there remains uncertainty regarding the potential risk of reversion to virulence during the treatment process. The deletion of GRA17 in the *T. gondii* type II ME49 strain leads to a partial reduction in the parasite’s virulence. Mice infected with the knockout strain survived at a dose of up to 1 × 10^5^ parasites, but died at a dose of 1 × 10^6^ parasites [70]. However, attenuated knockout strains of *T. gondii* still have the potential to form cysts, and the virulence and safety of these strains in immunocompromised hosts require further investigation [19, 103].

It is worth noting that pro-tumor effects of toxoplasmosis have also been suggested [33]. *Toxoplasma gondii* infection has been linked to an increased risk of glioblastoma [42]. The mortality rate from brain cancer is positively correlated with the seroprevalence of toxoplasmosis [97]. These studies, employing nested case-control designs, have reported that early IgG seropositivity to *T. gondii* is associated with an increased probability of subsequent cancer diagnosis. However, these findings remain contentious. First, the validity of the human evidence is severely limited: a single blood sample cannot establish the timing or stage of infection. The parasite exerts prolonged, stage-dependent effects on host cells. Nevertheless, the respective contributions of acute and chronic stages following seroconversion remain difficult to disentangle. Demonstrating a credible association would require decades of longitudinal follow-up, which is logistically and economically prohibitive. In addition, after *T. gondii* infects the human, it differentiates into bradyzoites and forms cysts. These cysts in the brain may induce chronic inflammation, which is a well-established carcinogenic risk factor [110]. Moreover, the absence of validated biomarkers for lifelong tissue cyst burden in humans means that current assays merely reflect cumulative exposure markers of past or recent acute infection; the impact of lifelong parasite persistence on tumorigenesis therefore remains undetermined [42]. Future research should assess exposure to chronic bradyzoite stage infection, which may be more relevant to the lifetime risk of tumor development. Consequently, existing studies can assert only a temporal association between *T. gondii* and increased cancer risk, not a causal relationship.

### Pre-existing *T. gondii* infection

Given that nearly one-third of the global population lives with chronic *T. gondii* infection, understanding how pre-existing infection shapes the efficacy of *T. gondii-*mediated cancer therapy is crucial. Evidence suggests that latent infection can enhance tumor resistance in murine models [41]. In mice previously infected with the Pru strain to establish latency, subsequent Δ*CPS* treatment remained effective, showing no reduction in its capacity to trigger a robust anti-tumor response in B16F10 tumors [4]. Similarly, in a study exploring the therapeutic effect of Δ*GRA17* on B16F10 tumors, the same latent infection modeling approach was conducted, and the results were consistent with those of the Δ*CPS* [115]. In mice with ovarian cancer and aged mice that had already established long-term immunity against *T. gondii* infection (induced by either the Δ*CPS* or Δ*OMP* vaccine), administering an additional dose of the Δ*CPS* or Δ*OMP* vaccine within the recommended vaccination interval significantly improved the survival rate of the ovarian cancer-bearing mice [36]. The effectiveness of this attenuated *T. gondii*-based immunotherapy appears to be independent of the host’s infection status, remaining unchanged in the presence of either chronic infection or pre-existing immunity to *T. gondii*.

The relationship between latent *T. gondii* infection and the central nervous system (CNS) remains controversial. It can form cysts in the brain for latent infection. Current research on pre-existing *T. gondii* infection also focuses on the CNS. Some studies show that a positive *T. gondii* serum rate can lead to cognitive impairment and memory decline in the elderly [9, 66]. Also, *T. gondii* infection aggravates the pathophysiological response and the degree of brain injury in mice after traumatic brain injury [6]. However, some studies have suggested that there is no correlation between Alzheimer’s disease and *T. gondii* seropositivity in the elderly, and pre-existing immunity can protect the brain from the consequences of subsequent damage [73, 100]. The reasons for these differences may lie in the fact that current studies are largely based on serological retrospective analyses and acute infection with *T. gondii* tachyzoites, and lack in-depth research on low-virulence chronic infection. Low-virulence strains can induce low-grade inflammation, encompassing both pro- and anti-inflammatory responses [11, 59], which prepares the brain for subsequent damage. On the contrary, pathogenic strains can lead to immunosuppression, rather than low-grade inflammation, which makes the brain ready for subsequent damage [99].

### Clinical translation difficulties

Research on the anti-tumor treatment of *T. gondii* is primarily based on experimental animal models, with a lack of clinical efficacy assessments. Laboratory mice are the most widely used animal models in *T. gondii-*related studies. Firstly, the immune response and prognosis induced by *T. gondii* infection are associated with the mouse strain. BALB/c mice exhibit stronger resistance to *T. gondii* infection compared to C57BL/6 mice. Previous studies have indicated that C57BL/6 mice are associated with a more robust immune response through the expression of MHC genes [14]. Hence, the choice of distinct preclinical models may result in variations in final clinical utility, thereby heightening the difficulty of clinical translation.

The shift from experimental research to clinical practice is chiefly impeded by species disparities between lab mice and humans. TLR11 and TLR12 receptors, innate sensors in mice for detecting *T. gondii* infection, boost IL-12 and IFN-γ production. However, humans lack the TLR12 receptor, and the TLR11 gene fails to trigger functional protein transcription [91, 104]. Therefore, the targets through which *T. gondii* enhances host immunity in experimental mice may not be applicable in humans. Organoid models circumvent these limitations. Generated *in vitro* through the self-organization of stem or tissue-specific cells, these three-dimensional “mini-organs” retain the parental tumor’s genomic and phenotypic heterogeneity, while restoring human immune components [65, 74]. Such systems faithfully recapitulate tumor biology and the efficacy of immunotherapies *in vitro*, yielding significant advances in bladder [55], gastrointestinal, and other solid malignancies, and enabling robust modeling of immune-checkpoint blockade [37]. They not only help us to better understand the complexity of tumors, but also provide powerful tools for the development of new treatment methods.

On the other hand, the use of *T. gondii* in cancer therapy faces several technical challenges due to the specific growth conditions of the parasite. The protozoan can only proliferate inside living tissue cells, and its *in vitro* cultivation must be conducted in culture flasks containing a monolayer of cells. This renders *T. gondii* less accessible than conventional pharmaceuticals, while also complicating its large-scale production and storage [17, 28]. The revival and cultivation of *T. gondii* required days in advance before its application in anti-tumor treatment could potentially delay therapeutic opportunities, which is highly impractical. Therefore, making *T. gondii* more readily available is a consideration for its application in cancer therapy [111]. Additionally, the optimal dosage and administration route for different *T. gondii* strains in various cancer treatments requires extended validation.

### Chronic inflammatory response

Differentiation of *T. gondii* tachyzoites into bradyzoites and their subsequent encystation establish chronic infection, yet tissue cysts and the enclosed bradyzoites are far from inert. Within cysts, bradyzoites continue low-level replication and aggregation, sustaining parasite persistence. Chronic carriage of these cysts elicits both humoral and cell-mediated immune responses that maintain active, lifelong parasitism, particularly within the CNS. This persistent low-grade inflammation can have dual effects: it may promote tumorigenesis through DNA damage and mutagenesis, but under certain conditions, such as strong immune activation induced by *T. gondii*, it can enhance immune surveillance and suppress tumor development [89]. Thus, the parasite exemplifies the dualistic and context-dependent nature of the inflammation–cancer axis.

Inflammation is crucial in tumorigenesis. Chronic inflammation due to *T. gondii*’s latent cyst-forming infection in immunocompromised individuals, along with cancer-induced immune system weakening, creates a tumor-favoring environment. Targeting the TME’s inflammatory pathway is an effective cancer treatment [93]. The balance of the inflammatory response matters in both anti-*Toxoplasma* and anti-tumor efforts. *Toxoplasma gondii* triggers the NF-κB signaling cascade in host cells, leading to the generation of IL-6, which plays a role in the host’s immune response to the parasite’s invasion [48]. The IL-6 family, particularly IL-6 and IL-11, are significantly elevated in many cancers, causing chronic inflammation and promoting tumor development [94]. The use of the anti-human IL-6 antibody drug cetuximab to inhibit the IL-6/STAT3 signaling pathway has a therapeutic effect on breast cancer [24]. In addition, in the later stage of *T. gondii* infection, the chronic inflammation mediated by Th2, which produces anti-inflammatory factors, such as IL-4 and IL-10, may further promote tumor growth [45].

**Figure 2:**
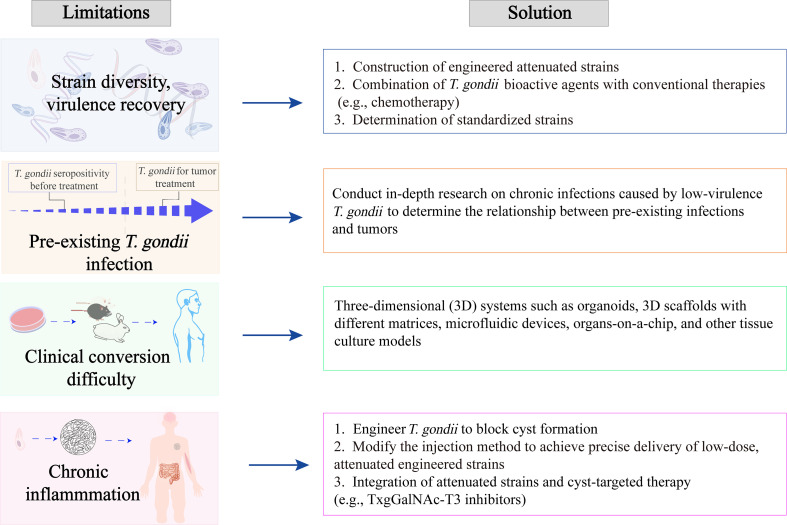
Factors that may limit the development of *T. gondii*’s anti-tumor effects.

Real-time monitoring of inflammatory cytokines during *T. gondii-*based cancer therapy allows treatment regimens to be adjusted promptly, which can mitigate the risks of cytokine storms and tumor-promoting effects; however, this approach may raise significant cost-effectiveness concerns. Therapeutic strategies directed against *T. gondii* cysts have laid the groundwork for antitumor research. Inhibitors of the mucin-type O-glycosyltransferase TxgGalNAc-T3 may weaken the cyst wall, enabling the immune system to eliminate cysts and latent infections [53]. However, whether such cyst-targeted drugs exert any impact on the functions of *T. gondii* tachyzoites remains to be further investigated. The characteristic of cyst-deficient strains to fail in forming persistent infections may enhance the safety and controllability during *T. gondii*-mediated tumor treatment. Deletion of the *T. gondii* cyst wall protein CST1 markedly reduces cyst burden and compromises cyst-wall integrity [95]. Likewise, genetic ablation of the cyst-development factors GRA4 and GRA6 profoundly diminishes the parasite’s cyst load [35]. Employing engineered parasites that either block cyst formation or combining attenuated strains with cyst-targeted therapies can preserve the antitumor efficacy of *T. gondii* while avoiding the pro-tumorigenic consequences of chronic inflammation (Fig. 2).

## New trends in the development of the anti-tumor effect of *T. gondii*

### Engineering *T. gondii* strain optimization strategies

Microbe-based cancer therapy is an extremely promising adjunct to conventional cancer treatment [101]. Nevertheless, conventional immunotherapy using *T. gondii* fails to ensure the exclusive invasion of tumor cells. It is likely to trigger systemic non-specific immune activation, which in turn may give rise to excessive inflammatory reactions and systemic toxic side effects. Given that the TME comprises a complex network of tumor cells, immune cells, fibroblasts, endothelial cells, and pericytes, achieving targeted invasion of tumor cells by *T. gondii* is of critical importance for both efficacy and safety. At present, although replication-deficient *T. gondii* strains like RH-Δ*CPS* or other strains have been developed, it remains uncertain whether these strains can specifically target tumor cells instead of other cells in the surrounding environment. Therefore, deeply analyzing the molecular mechanism by which *T. gondii* specifically targets tumor cells without invading other cells in the TME and minimizing off-target effects is the key to anti-tumor treatment with *T. gondii* [101, 107].

Optimizing *T. gondii* administration may improve tumor targeting. Compared with intravenous and intraperitoneal injection, intratumoral injection of Δ*CPS* and Δ*GRA17* strains can make the tumor subside, while intravenous and intraperitoneal injection can only slow down the growth and fail to eliminate the primary tumor [4, 115]. Intravenous injection, oral administration, and intraperitoneal injection provide the activation of systemic extensive immune response. Intratumoral injection is more about the local targeted killing ability in the tumor *in situ*. In the selection of treatment methods, combined treatment or a single scheme can be selected according to the tumor location, the patient’s immune ability, and other conditions, similar to the mode of action of oncolytic viruses [77].

It is a novel strategy for future anti-tumor application of *T. gondii* to contemplate incorporating linker molecules that directly target tumor cell-specific antigens into *T. gondii via* genetic engineering techniques. Currently, a strategy involving the display of functional single-chain variable fragments (scFvs) on the surface of *T. gondii* has been validated to target specific cells, while minimizing non-specific binding. Specifically, *T. gondii* that displays scFvs against the dendritic cell endocytic receptor DEC205 or the immune checkpoint PD-L1 on its surface exhibits significantly stronger binding ability to target cells than wild-type strains, with almost no off-target effects. Furthermore, when the *T. gondii* SAG1 coding sequence is fused with the anti-DEC205 scFv, the resulting construct exhibits enhanced functional activity in target binding [2].

The replication defective strain of *T. gondii* with the best engineering design avoids replication during treatment, and makes *T. gondii* only invade tumor cells in the TME through surface display technology, so it can more accurately target tumor cells and reduce non-specific immune response, which has become a new direction for the treatment of tumors with *T. gondii*.

### Exploration of anti-tumor bioactive agents derived from *T. gondii*

The exploration of bioactive agents with anti-tumor effects from *T. gondii* also holds potential application value, especially in activating the host’s innate immune response and enhancing the anti-tumor immune response. Based on this, the anti-tumor bioactive agents from *T. gondii* may have a broader scope of application and higher safety. *Toxoplasma gondii* profilin-like protein (TgPLP) serves as an immune adjuvant in the vaccination of mice with autologous whole-cell vaccine (AMV). Compared to AMV vaccination alone, the combination of TgPLP and AMV enhances the immune response, leading to smaller tumor size and improved survival rates [76]. The combined treatment of autoclaved *T. gondii* vaccine (ATV) and cancer immunomodulator cyclophosphamide (CP) for Ehrlich solid cancer exhibits a synergistic immunotherapeutic effect and anti-angiogenic effect with significant hypertrophy and hyperplasia of Kupffer cells and sinusoidal lymphocyte infiltration [49]. *Toxoplasma* Lysate Antigen (TLA) exhibits significant anti-tumor activity, capable of inhibiting tumor growth in tumor-bearing rats induced by methylcholanthrene, and by suppressing CD31 expression, it leads to a reduction in tumors in S180 tumor-bearing mice [63, 75]. Exosomes derived from *T. gondii* have been shown to activate the host’s innate immunity, significantly increasing the expression of IL-12, TNF-ɑ, and IFN-γ, thereby alleviating the immunosuppressive microenvironment and holding broad prospects in cancer therapy [111].

Although bioactive agents derived from *T. gondii* have limited efficacy and can only slow tumor growth rather than induce regression as live engineered strains do, inactivated *T. gondii* preparations may be more ethically acceptable for clinical use. When combined with other cancer therapies, they may produce complementary or synergistic effects. Research on *T. gondii*-driven anti-tumor bioactive agents is a cutting-edge yet complex field. It requires in-depth exploration of the interaction mechanisms between *T. gondii* and its host, as well as the identification of precise bioactive agents with anti-tumor activity. On this basis, high-throughput screening *via* bioinformatics prediction and CRISPR/Cas9 technology can be used to identify anti-tumor bioactive agents from *T. gondii*, including proteins, polypeptides, and polysaccharides, thereby discovering more candidates for anti-tumor bioactive agents.

### Engineered *T. gondii* as a drug carrier for brain tumors

Technologies that repurpose parasites into novel drug delivery vectors by leveraging their inherent characteristics have been studied extensively. For instance, helminth parasites have attracted increasing attention as live immunotherapeutic agents and *in vivo* biopharmaceutical factories. Notably, helminth infection triggers a Th2-type immune response, making it particularly suitable for the treatment of inflammatory diseases. Recently, a genetic engineering strategy for helminths has been proposed, in which genomic regions with high expression of exogenous secretory proteins (ESPs) are targeted to regulate ESP-encoding loci actively expressed in adult worms, thereby enabling the production of bioactive molecules [98]. A typical example is the oral administration of *Necator americanus* larvae, which can persist in the intestine, upregulate the levels of incretins such as GLP-1 and GIP, and significantly improve insulin resistance in patients with type 2 diabetes (T2DM) [27].

Drug delivery is often challenged by biological barriers that prevent molecules from reaching target sites. Interestingly, *T. gondii* has proven ability to penetrate the placental barrier, the blood-brain barrier (BBB), and the blood-testis barrier [79]. The migratory characteristics of *T. gondii* and its secretion system have been studied for their potential to directly deliver drug molecules to host cells, including those in the CNS, which are typically inaccessible to drug molecules. The BBB, which tightly regulates the entry and exit of various molecules into the CNS, is primarily composed of brain endothelial cells, basement membranes, tight junctions, astrocytes, and pericytes. It is virtually impermeable to most large biological molecules (>400 Da), thereby excluding almost all proteins [7]. Some drugs can permeate into the brain parenchyma through the blood-cerebrospinal fluid barrier. However, the flow rate of cerebrospinal fluid in the brain parenchyma is very low, which restricts the permeation of cerebrospinal fluid from blood vessels into the brain parenchyma [8, 69].

Engineered *T. gondii* has demonstrated significant potential in delivering therapeutic proteins to the brain, serving as a drug delivery vehicle for brain tumors. It can migrate to the CNS through transendothelial pathways, paracellular routes, and the “Trojan horse” mechanism. *Toxoplasma gondii* can hijack immune cells, using the “Trojan horse” strategy to evade host immunity and penetrate the BBB to invade the CNS [23]. The characteristic of *T. gondii* in manipulating immune cells to reach the CNS is beneficial for drug delivery to the brain *via* immune cells. Researchers have ingeniously created translational fusions of therapeutic proteins with *T. gondii* toxofilin and GRA16. This approach exploits the parasite’s endogenous secretory machinery, specifically the rhoptries and dense granules, to achieve precise delivery of therapeutic payloads into neurons by coupling *T. gondii* secretory proteins with functional biomolecules [13]. The discovery of additional *T. gondii* secreted proteins with anti-tumor efficacy to serve as carriers and their integration with therapeutic drugs, could constitute a strategy for further augmenting the therapeutic outcome of brain tumors.

### Combination of *T. gondii* with other anti-tumor therapies

The combination of *T. gondii* therapy with other anti-tumor therapies is a potentially novel treatment strategy, aiming to enhance the anti-tumor effect and reduce adverse reactions through the synergistic effect of different mechanisms, such as immune checkpoint inhibitors (ICB), chemotherapy, radiotherapy, and other immunotherapies (CAR-T cell therapy, tumor vaccines, *etc.*). The use of attenuated *T. gondii* can improve the sensitivity of tumors that fail to respond to ICB treatment, the combined therapy using the RHΔ*GRA17* strain and the blockade of immune checkpoint (PD-L1) makes TMB more sensitive to PD-L1 blocking therapy, which can remarkably prolong the survival rate of mice with melanoma, Lewis lung cancer, and colon adenocarcinoma, and suppresses tumor growth [115]. The combination therapy of NRTUA and anti-PD-1 antibody also induced a significant anti-tumor immune response and synergistically controlled tumor growth in PDAC tumor-bearing mice [3]. Combining *T. gondii* with chimeric antigen receptor CAR-T cells enhances target specificity and significantly boosts anti-tumor efficacy [29]. Co-treatment with GRA16 and the chemotherapeutic drug irinotecan blocks NF-κB activation by modulating the PP2A-B55/AKT/NF-κB signaling pathway, thereby enhancing the chemotherapeutic efficacy of irinotecan against non-small cell lung cancer cells [87].

The combination of *T. gondii* therapy and other anti-tumor therapies, using the synergistic effect of diverse mechanisms, augments the anti-tumor efficacy, potentially lessening the dosage and adverse effects of a single treatment modality, thereby affording patients a greater range of treatment alternatives and prospects. Nevertheless, the interaction mechanisms among the various therapies remain incompletely elucidated and demand in-depth exploration. Combining treatments may increase complexity and cost, requiring careful evaluation of the patient’s condition and response to ensure the best outcomes [58]. As understanding of *T. gondii* therapy and other anti-tumor treatments deepens, and clinical trials progress, this combined strategy may offer hope to more patients with cancer.

### Cost-effectiveness evaluation

Due to the lack of cost-benefit evaluation of *T. gondii* in the treatment of tumors, the cost-benefit analysis can only be carried out from the perspective of research and development of new tumor drugs. Therapy with *T. gondii* is a novel anti-tumor treatment that needs to demonstrate efficacy, safety, and cost-effectiveness compared to existing therapies. The cost of *T. gondii* treatment includes: i) research and development costs (R&D costs), which involve significant investments in drug screening, animal experiments, and clinical trials; there are as yet no standardized strains of *T. gondii* for tumor therapy, and research into its anti-tumor effects remains in its infancy. Extensive basic studies are therefore needed to clarify its mechanisms of action and refine treatment regimens, a task that will incur significant R&D expenditure. Moreover, in countries where parasite infection rates are low, national R&D funding may be limited [30]; ii) production costs, which cover raw materials, equipment, and quality control for large-scale manufacturing. As an intracellular parasite, *T. gondii* can only grow and replicate inside host cells. Laboratory cultivation of this parasite relies on cell culture techniques, which significantly increases the difficulty and cost of obtaining *T. gondii*; and iii) medical costs, including multiple treatment sessions, monitoring, and additional expenses for adverse reactions or complications. Different strains of *T. gondii* exhibit marked variations in both virulence and anti-tumor efficacy, necessitating personalized immunotherapy for different patients. The development of individualized delivery systems serves as a prime example of this need [16].

The cost-benefit analysis of *T. gondii*-based anti-tumor therapy must weigh multiple factors. As a major global health issue, cancer creates enormous demand for effective treatments. Cancer continues to impose a significant global health and economic burden, creating an urgent need for innovative and effective treatment options. If *T. gondii* therapy proves successful, it could complement existing cancer treatments, reduce patient and societal costs, and yield meaningful public health benefits. In short, the cost-benefit analysis of *T. gondii* anti-cancer therapy is a complex subject that requires a comprehensive assessment of all factors. As research and technology advance, we hope to evaluate its cost-effectiveness more precisely and provide patients with more effective, safe, and economical treatment options [109].

## Conclusion

*Toxoplasma gondii* is capable of infecting a wide range of hosts and has drawn increasing attention as a potential tool in cancer therapy. It exerts multiple anti-tumor effects through diverse mechanisms. Specifically, *T. gondii* can activate the innate and adaptive immune systems, mature dendritic cells and enhance antigen presentation ability, induce the production of key cytokines such as IL-12 and IFN-γ, and then activate immune cells to generate a strong Th1 immune response to kill tumor cells. In addition, the parasite can also change the immunosuppressive state of the TME, reduce inhibitory immune cells, increase activated immune cells, directly induce apoptosis of tumor cells, inhibit their proliferation, and inhibit tumor angiogenesis by regulating relevant signals. However, the use of *T. gondii* in cancer treatment faces many challenges. The parasite exhibits complex strain-specific characteristics, with substantial variation in virulence, infection patterns, and anti-tumor efficacy among different isolates. Moreover, some evidence suggests that *T. gondii* infection may contribute to tumorigenesis under certain conditions, such as in brain cancer. Clinical translation is further hindered by ethical concerns surrounding the use of wild-type strains, difficulties in extrapolating animal model results to humans, and the parasite’s requirement for living host cells, which complicates large-scale cultivation and application. Additionally, excessive inflammatory responses may paradoxically promote tumor progression. However, some new trends have emerged currently, including developing engineered strains to accurately target tumor cells, exploring the anti-tumor effects of its bioactive agents, using its barrier-penetrating properties as a drug carrier for brain tumors, attempting to combine with other anti-tumor therapies, and comprehensively evaluating cost-effectiveness, *etc.* Collectively, these advances provide new opportunities and renewed optimism for *T. gondii-*based cancer therapy.
